# Comparative Outcomes of Transcatheter Versus Surgical Aortic Valve Replacement in Moderate-Risk Patients With Aortic Stenosis: A Systematic Review of Clinical Trials

**DOI:** 10.7759/cureus.70268

**Published:** 2024-09-26

**Authors:** Zeeshan Ajmal, Zaeem Ur Rehman, Ayesha Ishtiaq, Hamdah Iftikhar, Mohammad M Khokhar, Bilal Khan, Ali Asad, Hannan Nasir, Syed Muhammad Athar, Ahmad Hassan, Hira Naveed

**Affiliations:** 1 Anesthesiology, Gulab Devi Hospital, Lahore, PAK; 2 Medicine, Islam Medical College, Sialkot, PAK; 3 Otolaryngology, Arif Memorial Teaching Hospital, Lahore, PAK; 4 Surgery, Quaid-e-Azam International Hospital, Islamabad, PAK; 5 Medicine and Surgery, Rai Medical College Sargodha, Sargodha, PAK; 6 Medicine, Shalamar Institute of Health Sciences, Lahore, PAK; 7 General Practice, Quaid-e-Azam International Hospital, Islamabd, PAK; 8 Medicine, Rai Medical College Sargodha, Sargodha, PAK; 9 General Surgery, Fazle Omer Hospital, Chiniot, PAK; 10 Anesthesiology, Gulab Devi Hospital, Lahore, Punjab, PAK; 11 General Practice, Shah Hussain Clinic, Faisalabad, PAK; 12 Medicine, Al-Aleem Medical College Lahore, Lahore, PAK; 13 Medicine, Gulab Devi Hospital Lahore, Lahore, PAK; 14 Biostatistics, Islamic International University, Islamabad, PAK; 15 Biostatistics, Gulab Devi Hospital, Lahore, PAK

**Keywords:** aortic stenosis, comparative outcomes, moderate-risk patients, surgical aortic valve replacement (savr), transcatheter aortic valve replacement

## Abstract

Aortic stenosis (AS) is a prevalent condition among the elderly, characterized by the narrowing of the aortic valve, which, if untreated, can lead to heart failure and decreased quality of life in terms of reduced activity and high mortality in one to two years. Surgical aortic valve replacement (SAVR) has long been the standard treatment for AS. However, it poses significant risks, particularly in older patients with comorbidities. In recent years, transcatheter aortic valve replacement (TAVR) has emerged as a less invasive alternative and is increasingly used in low- and moderate-risk patients. This review seeks to assess the comparative outcomes of TAVR and SAVR in patients with moderate-risk AS. A systematic review was conducted in accordance with PRISMA guidelines, focusing on randomized controlled trials (RCTs) that compared TAVR and SAVR in this patient population. Also, the review included three major RCTs: PARTNER 2, UK TAVI, and DEDICATE. We analyzed the key outcomes of TAVR and SAVR, such as mortality, reintervention rates, complications (such as myocardial infarction, prosthetic valve endocarditis, and pacemaker implantation), and reintervention rates, to evaluate the relative efficacy and safety of TAVR and SAVR. The analysis included data from 4,359 patients across the three trials. TAVR demonstrated a lower all-cause mortality in two of the three trials, with an overall trend favoring TAVR in terms of survival. However, TAVR was associated with a higher incidence of prosthetic valve endocarditis, a greater need for pacemaker implantation, and more frequent reinterventions compared to SAVR. In conclusion, the findings suggest that TAVR may be a better option for moderate-risk AS patients, offering higher survival rates and a less invasive recovery process. While TAVR carries increased risks of endocarditis and pacemaker dependency, its overall benefits, particularly in terms of lower mortality and improved patient outcomes, make it a preferable option over SAVR for many patients. However, acknowledging potential limitations such as variations in trial design and differences in patient populations would indeed provide a more comprehensive perspective. Further research and long-term follow-up are essential to confirm these findings and refine patient selection criteria.

## Introduction and background

Aortic stenosis (AS) is one of the most prevalent and serious valvular heart diseases in old age population and involves progressive narrowing of the aortic valve opening, which obstructs blood flow from the left ventricle to the aorta [1]. If not treated, this condition causes increased cardiac workload, which leads to left ventricular hypertrophy and, eventually, heart failure [2]. In symptomatic patients with medically treated moderate-to-severe AS, the mortality rate is approximately 25% at one year and 50% at two years following the onset of symptoms. [2]. The incidence of AS increases with age, and it is estimated that 2% to 7% of people over the age of 65 suffer from this condition, making it a major public health concern [3]. The natural history of untreated severe AS is poor, with symptomatic patients having a survival rate as low as 50% at two years and 20% at five years, highlighting the importance of early intervention [4].

Historically, the definitive treatment for symptomatic AS has been surgical aortic valve replacement (SAVR), which has been shown to significantly improve patient survival and quality of life [5]. SAVR is the surgical removal of a diseased aortic valve and replacement with a prosthetic valve, either mechanical or bioprosthetic [6]. While SAVR has been the gold standard treatment for decades, it carries significant perioperative risks, especially in elderly patients with significant comorbidities [6]. These risks include, but are not limited to, stroke, bleeding, infection, and a prolonged recovery period [2,6]. To address the limitations of SAVR, especially among high-risk and inoperable patients, transcatheter aortic valve replacement (TAVR) was developed as a less invasive option [7]. TAVR is performed using a catheter-based approach, which enables the implantation of a new valve within the diseased native valve without the need for open-heart surgery [7]. Since its inception, TAVR has transformed the management of AS, providing a viable treatment option for patients previously considered unsuitable for surgery [8]. TAVR's success has led to its widespread use in intermediate-risk patients, as evidenced by several key randomized controlled trials (RCTs) [8,9]. The PARTNER (Placement of Aortic Transcatheter Valves) trials, especially PARTNER 2, had been crucial in determining the efficacy and safety of TAVR in intermediate-risk patients. The PARTNER 2 trial found that TAVR was not inferior to SAVR in terms of the primary composite endpoint of death from any cause or disabling stroke at two years [10]. This trial also demonstrated the potential benefits of TAVR, such as shorter hospital stays and faster recovery times, which are especially beneficial to elderly patients. The DEDICATE trial investigated the role of TAVR in a broader patient population, including those at moderate surgical risk, with similar findings [11]. Another groundbreaking trial concluded that transcatheter aortic valve implantation (TAVI) was non-inferior to SAVR in terms of all-cause mortality at one year [12].

Even though it has a minimally invasive nature and expanding indications, TAVR also has some challenges in terms of widespread adoption. Several studies have expressed concern about the increased incidence of specific complications associated with TAVR, such as prosthetic valve endocarditis, permanent pacemaker implantation, and a higher rate of reintervention compared to SAVR [13,14]. Prosthetic valve endocarditis, a serious and often fatal infection of the valve, has been reported more frequently in TAVR patients, possibly due to the presence of more foreign material and residual valve tissue after the procedure [15]. Furthermore, TAVR patients are more likely to require permanent pacemaker implantation, which could be attributed to the valve's positioning and proximity to the conduction system [13]. Another active area of research is the long-term durability of TAVR valves, especially given that the procedure is increasingly used in younger, lower-risk patients with longer life expectancies [15]. While early-generation TAVR devices have demonstrated promising durability for up to five years, there are little data beyond this time frame, and concerns about structural valve deterioration necessitate careful patient selection and ongoing research [9,11]. Performing TAVR safely requires a high level of expertise and may necessitate the support of an experienced proctor, particularly in cases in which the operating team has limited experience. The complexity of the procedure and potential complications highlight the importance of skill acquisition through sufficient practice and mentorship. Proctor-assisted procedures can help ensure patient safety and optimal outcomes, especially during the early stages of a team's learning curve.

Given the complexities of AS treatment and the evolving role of TAVR, it is essential to continue comparing the outcomes of TAVR and SAVR across different patient populations. In this review, we aim to compare the outcomes of TAVR and SAVR in moderate-risk patients to determine the most effective and safe intervention modality.

## Review

Methodology

Search Strategy and Study Design

**Figure 1:**
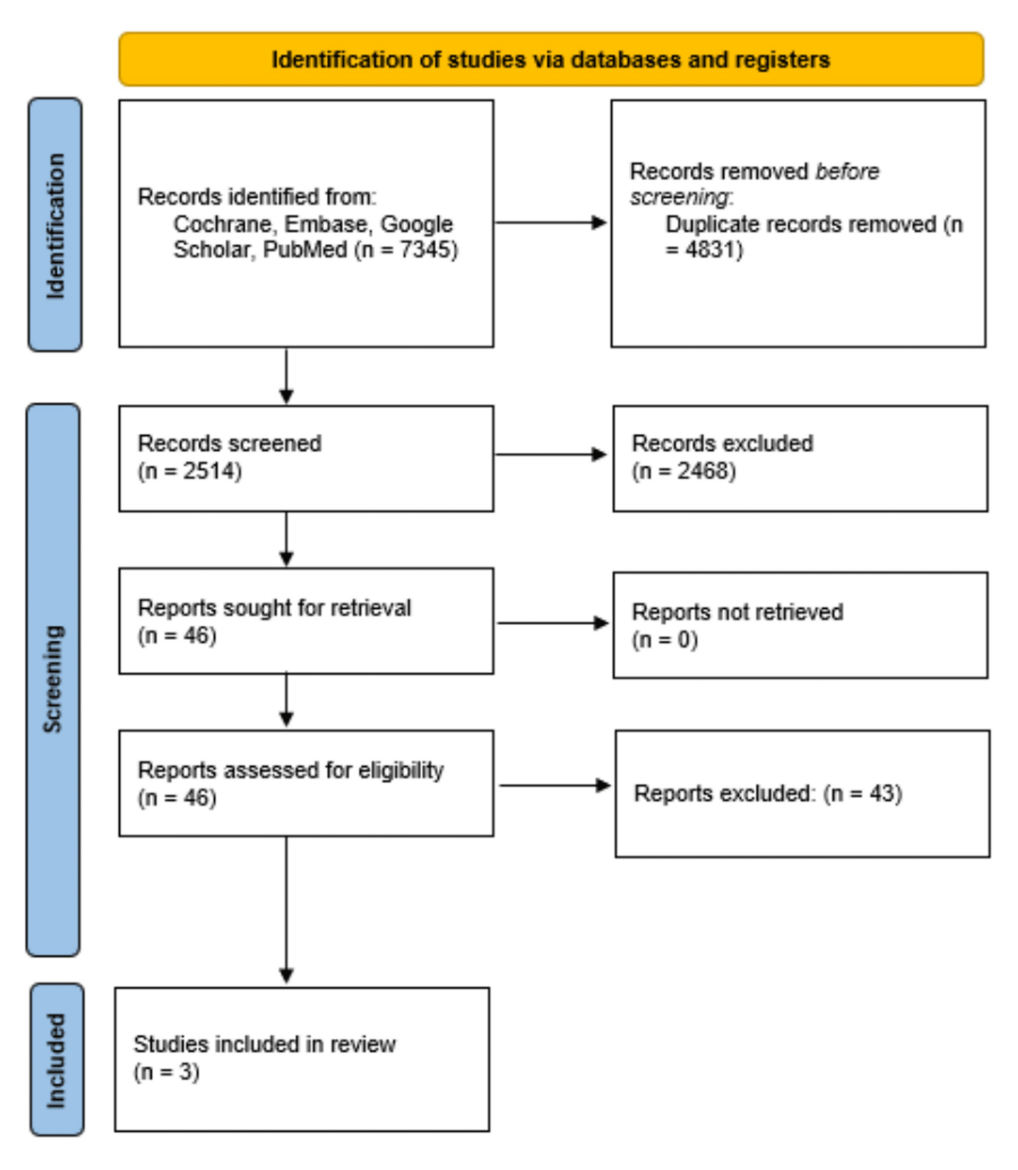
PRISMA flow diagram of study selection PRISMA, Preferred Reporting Items for Systematic Reviews and Meta-Analyses

This review followed the Preferred Reporting Items for Systematic Reviews and Meta-Analyses (PRISMA) guidelines for conducting a systematic review and analysis of RCTs. A comprehensive search strategy was developed using Boolean operators to identify studies comparing TAVR and SAVR in patients with symptomatic AS at moderate surgical risk. The search combined keywords using the operators "AND" and "OR" as follows: ("Aortic Stenosis" OR "Aortic Valve Stenosis" OR "AS" OR "Aortic Valve Disease") AND (("Transcatheter Aortic Valve Replacement" OR "TAVR" OR "TAVI" OR "Transcatheter Aortic Valve Implantation" OR "Catheter-Based Valve Replacement") OR ("Surgical Aortic Valve Replacement" OR "SAVR" OR "Open Surgical Valve Replacement" OR "Aortic Valve Surgery")) AND ("Moderate Risk" OR "Intermediate Risk" OR "Moderate Surgical Risk" OR "STS Score" OR "EuroSCORE") AND ("Mortality" OR "All-Cause Mortality" OR "Myocardial Infarction" OR "Prosthetic Valve Endocarditis" OR "Pacemaker Implantation" OR "Reintervention"). A comprehensive search was conducted across Cochrane, Embase, Google Scholar, and PubMed, yielding a total of 7,345 records. After removing 4,751 duplicate records, 2,514 unique records remained for screening. Following the screening of titles and abstracts, 2,468 records were excluded based on relevance. The remaining 46 reports were sought for full-text retrieval and were subsequently assessed for eligibility. After full-text screening, 43 reports were excluded for not meeting the inclusion criteria. Ultimately, three studies were included in the final systematic review (Figure 1). The study included three major RCTs: the PARTNER 2 trial (2020) [10], the UK TAVI trial (2022) [11], and the DEDICATE trial (2024) [12]. These trials were selected based on their relevance to the study's goal, which was to compare the clinical outcomes of TAVR and SAVR in patients with symptomatic AS at moderate surgical risk.

Eligibility Criteria

Studies were included based on the following criteria: (1) RCT design, (2) inclusion of patients with symptomatic AS classified as moderate surgical risk, (3) comparison of TAVR versus SAVR, and (4) availability of data on primary and secondary outcomes of interest. Exclusion criteria were non-randomized studies, trials with a different focus (e.g., high-risk or low-risk surgical patients), and studies without adequate outcome reporting.

Data Extraction and Outcome Measures

Data extraction was performed independently by four reviewers using a standardized form. Discrepancies were resolved through discussion, and a first author was consulted if needed. Extracted data included baseline demographic and clinical characteristics, procedural details, and follow-up outcomes. The primary outcome of interest was all-cause mortality at one year. Secondary outcomes included stroke, myocardial infarction (MI), prosthetic valve endocarditis, permanent pacemaker implantation, and the need for aortic valve reintervention. These outcomes were selected based on their clinical relevance and consistent reporting across the trials.

Quality Assessment

The quality of the included studies was assessed using the Cochrane Risk of Bias tool. Each trial was evaluated for potential biases related to randomization, blinding, incomplete outcome data, selective reporting, and other sources of bias. The overall risk of bias was judged as low, moderate, or high for each study, and this assessment was considered when interpreting the results.

Statistical Analysis

Descriptive statistics were used to summarize the baseline characteristics of the study populations, with continuous variables reported as means with standard deviations and categorical variables as counts with percentages. Comparative analyses between the TAVR and SAVR groups were conducted using odds ratios (ORs) with 95% confidence intervals (CIs) for each outcome. Subgroup analyses were conducted to explore the impact of specific patient characteristics (e.g., age, STS risk score) on outcomes. The analyses were conducted with alternative statistical models to ensure consistency of findings.

Ethical Considerations

This analysis used de-identified data from previously published trials, and thus no new ethical approval was required. Each original trial had received appropriate ethical clearance from the relevant institutional review boards and adhered to the Declaration of Helsinki. Patient consent for inclusion in the original studies was obtained in accordance with standard clinical research protocols.

Results

This analysis included data from three RCTs: PARTNER 2 (2020) [10], UK TAVI (2022) [11], and DEDICATE (2024) [12]. These trials included a total of 4,359 patients with symptomatic AS, and all of them were classified as being at moderate surgical risk. Of these patients, 2,170 were allocated to TAVR and 2,189 to SAVR. The follow-up duration for PARTNER 2 was five years, while it was one year for UK TAVI and DEDICATE trials.

The mean age of the patient population across all studies was 79 ± 5.8 years. Gender distribution revealed a majority of male participants, with 2,397 males enrolled. The TAVR group had 1,187 male patients, while the SAVR group had 1,210 male participants. The mean Society of Thoracic Surgeons (STS) risk score, reflecting the surgical risk profile, averaged 3.4 across all studies, with specific scores of 5.8, 2.6, and 1.8 for TAVR patients in the PARTNER 2, UK TAVI, and DEDICATE trials, respectively, indicating variability in patient risk profiles among the studies.

Clinical characteristics showed that the majority of patients had significant comorbidities. The prevalence of New York Heart Association (NYHA) Class III or IV symptoms was notably high, with 782, 184, and 323 TAVR patients affected in the PARTNER 2, UK TAVI, and DEDICATE trials, respectively. Similarly, coronary artery disease was prevalent, particularly in the PARTNER 2 trial, where 700 TAVR patients were affected. The DEDICATE trial reported a high prevalence of hypertension, with 593 TAVR patients diagnosed, although these data were not reported for the PARTNER 2 trial. Diabetes mellitus was another common comorbidity, affecting a substantial proportion of patients, with 381 TAVR patients in the PARTNER 2 trial. Left ventricular ejection fraction (LVEF) was comparable between the TAVR and SAVR groups across the trials, with mean values ranging from 56.2% to 57.8% in TAVR patients and 56.2% to 57.7% in SAVR patients. Table 1 shows baseline characteristics and comorbidities.

**Table 1:** Study and Patient Characteristics TAVR, transcatheter aortic valve replacement; SAVR, surgical aortic valve replacement; STS, Society of Thoracic Surgeons; NYHA, New York Heart Association

Trial	PARTNER 2 [10]	UK TAVI [11]	DEDICATE [12]
Year	2020	2022	2024
Follow-up	5 years	1 year	1 year
TAVR (n)	1011	458	701
SAVR (n)	1021	455	713
Male (n)			
TAVR (n)	548	247	392
SAVR (n)	560	242	408
STS mean score			
TAVR (n)	5.8	2.6	1.8
SAVR (n)	5.8	2.7	1.9
BMI			
TAVR (n)	28.6	27.1	25.3
SAVR (n)	28.3	27.3	28.1
NYHA class III or IV			
TAVR (n)	782	184	323
SAVR (n)	776	204	325
Coronary artery disease			
TAVR (n)	700	133	238
SAVR (n)	679	145	270
Hypertension			
TAVR (n)	NR	328	593
SAVR (n)	NR	327	621
Diabetes mellitus			
TAVR (n)	381	107	237
SAVR (n)	349	111	233
LVEF (%)			
TAVR (n)	56.2 ± 10.8	57 ± 7.2	57.8 ± 9.8
SAVR (n)	56.2 ± 10.8	57 ± 8.1	57.7 ± 9.3
Permanent pacemaker			
TAVR (n)	118	31	37
SAVR (n)	123	29	35
Myocardial infarction			
TAVR (n)	185	43	36
SAVR (n)	179	40	53
Atrial fibrillation			
TAVR (n)	313	110	201
SAVR (n)	359	110	195

The primary outcome of interest, all-cause mortality, was reported in all three trials. A total of 964 (22.11%) deaths were reported in three trials, with 806 (39.66%) deaths in PARTNER 2 trial after five years of follow-up. UK TAVI and DEDICATE trial reported 51 (5.58%) and 107 (7.5%) deaths after one year of follow-up. In the PARTNER 2 trial, there were 436 deaths (43.12%) in the TAVR group and 370 deaths (36.2%) in the SAVR group. In contrast, the UK TAVI and DEDICATE trials reported 21 deaths (4.58%) and 37 deaths (5.27%) in the TAVR group, respectively, while they reported 30 deaths (6.5%) and 70 deaths (9.81%), respectively, in the SAVR group. Table 2 shows detailed mortality and post-procedure complications.

**Table 2:** Summary of post-procedural complications TAVR, transcatheter aortic valve replacement; SAVR, surgical aortic valve replacement

Trial	PARTNER 2 [10]		UK TAVI [11]		DEDICATE [12]	
	N	OR (95% CI)	N	OR (95% CI)	N	OR (95% CI)
All-cause mortality						
TAVR (n)	436	1.09 (0.95-1.25)	21	0.69 (0.38 to 1.26)	37	0.53 (0.35-0.79)
SAVR (n)	370		30		70	
Stroke						
TAVR (n)	128	1.15 (0.89-1.49)	24	1.98 (0.95 to 4.11)	20	0.61 (0.35-1.06)
SAVR (n)	107		12		33	
Myocardial infarction						
TAVR (n)	84	1.26 (0.91-1.75)	6	1.17 (0.33 to 4.20)	7	0.51 (0.20-1.19)
SAVR (n)	62		5		15	
Prosthetic valve endocarditis						
TAVR (n)	30	1.46 (0.82-2.60)	5	2.46 (0.54 to 11.17)	4	2.44 (0.87-8.15)
SAVR (n)	19		2		6	
Permanent pacemaker implantation						
TAVR (n)	138	1.20 (0.94-1.54)	65	2.05 (1.43 to 2.94)	82	1.81 (1.27-2.61)
SAVR (n)	113		33		47	
Aortic valve reintervention						
TAVR (n)	21	3.28 (1.32-8.13)	10	1.98 (0.72 to 5.42)	4	1.70 (0.38-9.78
SAVR (n)	6		5		2	

**Figure 2:**
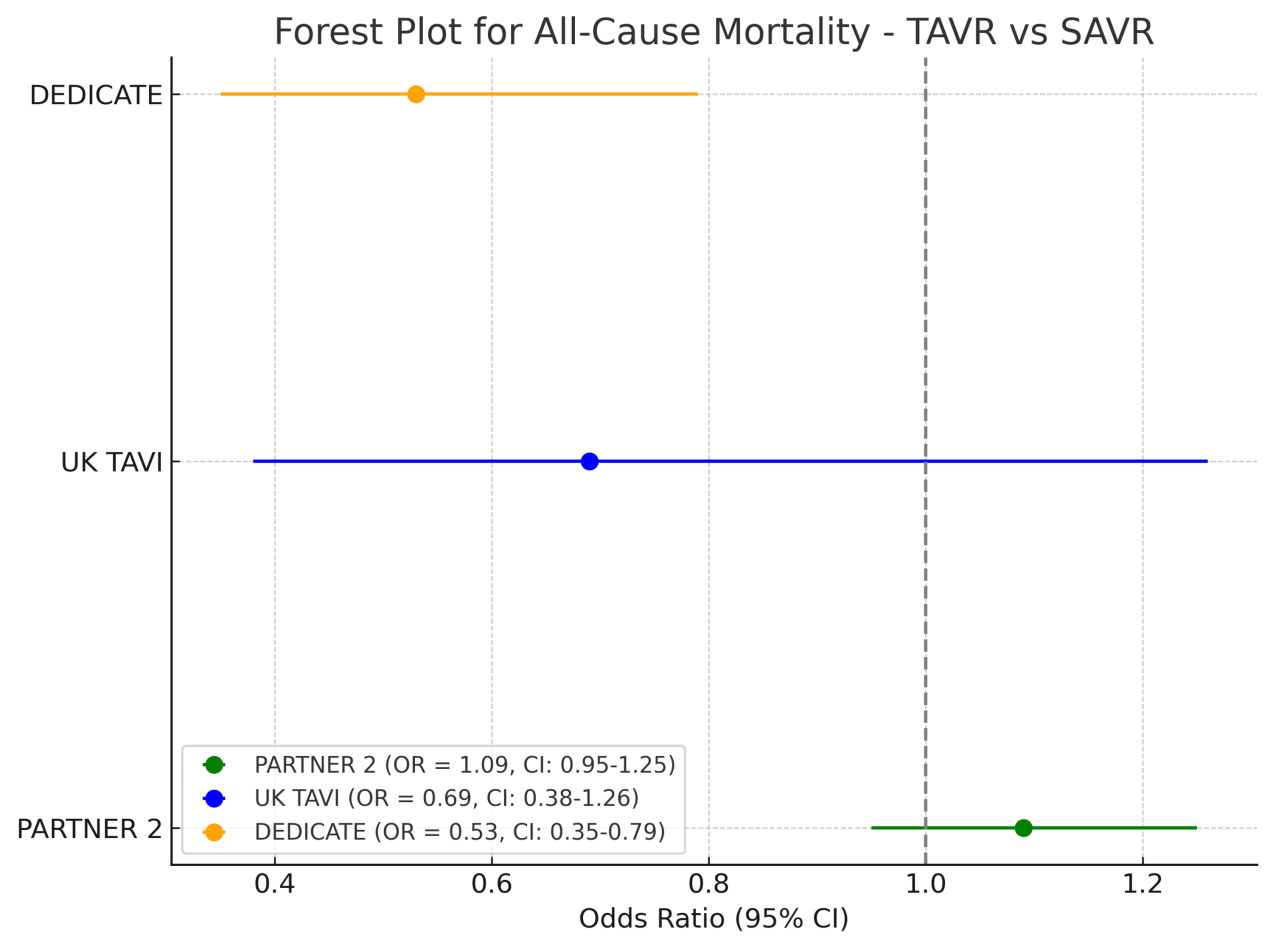
Risk estimates of all-cause mortality for transcatheter aortic valve replacement versus surgical aortic valve replacement TAVR, transcatheter aortic valve replacement; SAVR, surgical aortic valve replacement

The analysis revealed varying odds ratios (ORs) for all-cause mortality across the three trials. In the PARTNER 2 trial, the SAVR group exhibited a slightly lower risk of all-cause mortality (OR 1.09 (0.95-1.25) compared to TAVR. In contrast, the UK TAVI and DEDICATE trials demonstrated a trend toward lower all-cause mortality in the TAVR group, with ORs of 0.69 (95% CI 0.38-1.26) and 0.53 (95% CI 0.35-0.79), respectively (Figure 2). For MI, the PARTNER 2 trial suggested a higher risk associated with TAVR compared to SAVR (OR 1.26, 95% CI 0.91-1.75). The UK TAVI trial showed a similar trend, though with a lower OR of 1.17 (95% CI 0.33-4.20). Interestingly, the DEDICATE trial demonstrated a reduced risk of MI with TAVR (OR 0.51, 95% CI 0.20-1.19) relative to SAVR. The incidence of prosthetic valve endocarditis was higher in the TAVR group across all trials. The PARTNER 2 trial reported an OR of 1.46 (95% CI 0.82-2.60), while the UK TAVI and DEDICATE trials indicated even higher risks, with ORs of 2.46 (95% CI 0.54-11.17) and 2.44 (95% CI 0.87-8.15), respectively. Permanent pacemaker implantation rates were consistently higher in the TAVR group across the three trials. In the PARTNER 2 trial, the OR was 1.20 (95% CI 0.94-1.54). The UK TAVI trial reported a more pronounced increase (OR 2.05, 95% CI 1.43-2.94), while the DEDICATE trial showed a similarly elevated risk (OR 1.81, 95% CI 1.27-2.61). The need for aortic valve reintervention was notably higher in the TAVR group, particularly in the PARTNER 2 trial (OR 3.28, 95% CI 1.32-8.13). The UK TAVI and DEDICATE trials also indicated an increased risk of reintervention with TAVR, with ORs of 1.98 (95% CI 0.72-5.42) and 1.70 (95% CI 0.38-9.78), respectively.

All three trials exhibited a low risk of bias across all domains. Therefore, the results of these trials were considered reliable and robust for comparative analysis. Table 3 shows a detailed risk-of-bias assessment.

**Table 3:** Risk-of-bias assessment of included studies

Study Name	Bias Arising From the Randomization Process	Bias Due to Deviations from Intended Interventions	Bias Due to Missing Outcome Data	Bias in Measurement of the Outcome	Bias in Selection of the Reported Results	Overall Risk of Bias
PARTNER 2 [10]	Low risk	Low risk	Low risk	Low risk	Low risk	Low risk
UK TAVI [11]	Low risk	Low risk	Low risk	Low risk	Low risk	Low risk
DEDICATE [12]	Low risk	Low risk	Low risk	Low risk	Low risk	Low risk

Discussion

This systematic review provides a comprehensive comparison of TAVR and SAVR in patients with symptomatic AS at moderate surgical risk. The analysis included data of more than 4,000 patients from three key RCTs. The primary outcome of interest was all-cause mortality at one year, with secondary outcomes including rates of MI, prosthetic valve endocarditis, permanent pacemaker implantation, and aortic valve reintervention.

The primary analysis and interpretation of all-cause mortality at one year showed a complex picture. In the UK TAVI and DEDICATE trials, TAVR demonstrated a trend towards lower mortality compared to SAVR, with odds ratios (ORs) of 0.69 (95% CI 0.38-1.26) and 0.53 (95% CI 0.35-0.79), respectively. This finding is consistent with earlier studies, such as the PARTNER 2 trial, which demonstrated that TAVR was not inferior to SAVR in intermediate-risk patients, with comparable mortality or disabling stroke at two years [10]. Similar results were reported in the SURTAVI trial, where TAVR showed non-inferiority to SAVR in intermediate-risk patients in terms of all-cause mortality and stroke [16]. The lower mortality observed in the TAVR group in the UK TAVI and DEDICATE trials may be attributed to the procedure being less invasive, leading to reduced perioperative risks, shorter duration of hospital stays, and quicker recovery times [11,12]. These benefits are particularly notable in elderly patients with multiple comorbidities, who are at higher risk of complications following open-heart surgery.

In contrast, the PARTNER 2 trial in our analysis reported a slightly higher risk of all-cause mortality in the TAVR group compared to SAVR (OR 1.09, 95% CI 0.95-1.25). This result highlights the variability in outcomes observed across different studies and patient populations [10]. Several factors can influence this variability such as operator experience, patient selection, and the type of TAVR device used [17,18]. The analysis of MI outcomes showed that TAVR was associated with a slightly elevated risk of MI compared to SAVR in both the PARTNER 2 and UK TAVI trials, with ORs of 1.26 (95% CI 0.91-1.75) and 1.17 (95% CI 0.33-4.20), respectively [10,11]. These findings align with previous studies that have shown a trend towards a higher incidence of MI in TAVR patients, particularly in the early post-procedural period [19]. Different procedural factors, such as valve positioning and coronary artery obstruction, maybe related to the increased risk of MI, which is observed more commonly in TAVR than in SAVR [20]. Interestingly, in comparison, the DEDICATE trial in our analysis reported a lower risk of MI with TAVR compared to SAVR (OR 0.51, 95% CI 0.20-1.19) [12]. This result suggests that the risk of MI with TAVR may vary depending on selection of patient population and procedural techniques. Further research and studies are needed to clarify the long-term impact of TAVR on cardiovascular outcomes, particularly in candidates with significant coronary artery disease [21]. The differences in patient demographics and expertise between the PARTNER and DEDICATE trials highlight important factors that should be considered in comparing their outcomes. While the PARTNER trial targeted moderate to high-risk patients with a mean age of 80 years, the DEDICATE trial focused on low- to intermediate-risk patients, primarily those aged 65 and older. This variation in patient risk profiles and age groups may account for differences in procedural outcomes and complication rates. Moreover, since the PARTNER trial was conducted earlier, the expertise and experience of clinicians in TAVI could have evolved by the time of the DEDICATE trial, potentially influencing the procedural success and safety profiles. These distinctions are critical when interpreting the results and generalizing findings to different patient populations.

One of the main concerns associated with TAVR is the increased risk of prosthetic valve endocarditis. Our analysis found that TAVR resulted in higher rates of endocarditis across all three trials, with ORs ranging from 1.46 (95% CI 0.82-2.60) in the PARTNER 2 trial to 2.46 (95% CI 0.54-11.17) in the UK TAVI trial [11]. These findings are consistent with previous studies, which have reported higher rates of endocarditis in TAVR patients compared to SAVR [22,23]. The elevated risk may be attributed to different factors such as residual native valve tissue, foreign material associated with the TAVR device, and potential challenges in achieving a complete seal during deployment of the valve [24]. Another noteworthy complication observed with TAVR is the need for permanent pacemaker implantation. Our analysis found that TAVR was associated with a higher incidence of pacemaker implantation compared to SAVR, particularly in the UK TAVI and DEDICATE trials [11,12], where the ORs were 2.05 (95% CI 1.43-2.94) and 1.81 (95% CI 1.27-2.61), respectively. This observation in our analysis is in line with previous studies, which have demonstrated that TAVR is associated with a higher risk of conduction disturbances, which later translates into the need for a permanent pacemaker [25]. The mechanism involved in this complication is thought to be related to the proximity of the aortic valve to the conduction system, which can be disrupted during valve implantation [26]. The need for reintervention is another critical outcome when comparing TAVR and SAVR. Our analysis indicated that TAVR was associated with a higher risk of reintervention, particularly in the PARTNER 2 trial (OR 3.28, 95% CI 1.32-8.13). This result aligns with previous studies that have raised concerns about the long-term durability of TAVR valves, particularly in younger patients with longer life expectancies [27]. Factors that may contribute to the increased risk of reintervention in TAVR patients include valve mispositioning, paravalvular leak, and structural valve deterioration over time [28]. However, it is important to note that the risk of reintervention with TAVR has decreased with the introduction of newer-generation devices, which have improved design features and elements and have achieved better procedural success rates [29,30]. Ongoing research and long-term follow-up studies are needed to better understand the durability of TAVR valves and to develop strategies over time to minimize the risk of reintervention.

Overall, the findings of this systematic review highlight the complex decision-making process involved when selecting the appropriate treatment for patients with symptomatic AS at moderate surgical risk. While TAVR offers certain benefits over SAVR, mainly in terms of lower mortality in some trials, it is also associated with a higher risk of specific complications, such as prosthetic valve endocarditis and pacemaker implantation. These results highlight the importance of individualized patient selection and the need for close monitoring of the patient after procedure to reduce the risks associated with TAVR. Future research should focus on improving patient selection criteria to identify those who are most likely to benefit from TAVR while minimizing the risk of complications at the same time. Additionally, long-term and robust studies are needed to assess the durability of TAVR valves and to determine the optimal management strategies for patients who may require reintervention. As the indications for TAVR continue to expand, it is important to maintain a balanced perspective on the benefits and risks of this procedure, particularly in younger and lower-risk patients. Acknowledging the limitations of this review is essential for providing a balanced perspective. Potential biases in the included trials, such as differences in patient selection criteria, procedural techniques, and the level of expertise among clinicians, may have influenced the outcomes. Additionally, variations in how the procedures were conducted across different studies could introduce heterogeneity that impacts the comparability of results. By recognizing these limitations, we aim to present a more nuanced interpretation of the findings and their applicability to broader clinical practice.

## Conclusions

This systematic review demonstrates that TAVR is non-inferior to SAVR in moderate-risk patients with AS. The data suggest that TAVR offers comparable outcomes in terms of survival while providing the additional benefits of a less invasive procedure, leading to quicker recovery and reduced hospital stays. However, the potential for increased risks, such as prosthetic valve endocarditis and the need for permanent pacemaker implantation, should not be overlooked. These considerations highlight the importance of careful patient selection, close post-operative monitoring, and a tailored approach to treatment. A heart team approach plays an exceptional role in optimizing outcomes by ensuring that individualized patient decisions are made with the input of a multidisciplinary team, considering both the clinical complexity and personal circumstances of each patient. This collaborative decision-making process is critical for balancing the benefits of TAVR’s less invasive nature with the potential risks. While TAVR is a promising alternative to SAVR, particularly for patients who may not tolerate surgery well, the choice between the two should be carefully individualized. Further research is essential to refine these considerations and continue improving outcomes for this patient population.
